# Views of Somali women and men on the use of faith-based messages promoting breast and cervical cancer screening for Somali women: a focus-group study

**DOI:** 10.1186/s12889-017-4182-2

**Published:** 2017-03-20

**Authors:** Rebekah Pratt, Sharif Mohamed, Wali Dirie, Nimo Ahmed, Michael VanKeulen, Huda Ahmed, Nancy Raymond, Kola Okuyemi

**Affiliations:** 1 Program in Health Disparities Research, Department of Family Medicine and Community Health, University of Minnesota, Minneapolis, USA; 2 Islamic Civil Society of America, Minneapolis, USA; 3 Department of Family Medicine and Community Health, University of Minnesota, Minneapolis, USA; 4 Open Path Resources, Minneapolis, USA; 5 Program in Health Disparities Research, University of Minnesota, Minneapolis, USA; 6 Powell Center for Women’s Health, Department of Psychiatry, University of Minnesota, Minneapolis, USA

## Abstract

**Background:**

Screening rates for breast and cervical cancer for Muslim women in the United States are low, particularly for first-generation immigrants. Interpretations of the Muslim faith represent some of the barriers for breast and cervical cancer screening. Working to understand how faith influences breast and cervical screening for Somali women, and working with the community to identify and utilize faith-based assets for promoting screening, may lead to life-saving changes in screening behaviors.

**Methods:**

We partnered with an Imam to develop faith-based messages addressing the concerns of modesty and predetermination and promoting cancer testing and screening. A total of five focus groups were convened, with 34 Somali women (three groups) and 20 Somali men (two groups). Each focus group first discussed participant views of breast and cervical cancer screening in general and then viewed and discussed video clips of the Imam delivering the faith-based messages.

**Results:**

Both Somali women and men had an overwhelmingly positive response to the faith-based messages promoting breast and cervical cancer screening. The faith-based messages appeared to reinforce the views of those who were already inclined to see screening positively, with participants describing increased confidence to engage in screening. For those who had reservations about screening, there was feedback that the faith-based messages had meaningfully influenced their views.

**Conclusions:**

Somali immigrant women and men found faith-based messages addressing topics of predestination and modesty and encouraging the use of screening and treatment to be both acceptable and influential. Faith can play an important role as an asset to promote breast and cervical cancer screening, and there may be substantial benefits to adding faith-based messaging to other interventions that focus on improving screening uptake. This may help to address health disparities for Somali women in this area.

## Background

Screening rates for breast and cervical cancer are low for Muslim women in the United States (US) [1], particularly for first-generation immigrants [2, 3]. Approximately half of Somali women have received appropriate cervical cancer screening [4], and 61% have been screened for breast cancer [5]. However Somali women living in areas of economic disadvantage may have much lower rates, as low as 8% for mammography according to Minnesota Community Measures, and self-reports may be subject to overestimation. There is a need to address disparities in breast and cervical screening for Somali immigrant women, and thereby reduce the burden of late detection and increase access to both preventive care and treatment.

Many barriers put Somali women at particularly high risk for not engaging in breast and cervical cancer screening, including limited health literacy [6] and a general lack of knowledge about cancer [7, 8]. In addition, there are concerns around female circumcision [8, 9], such as whether the risk of pain with cervical cancer screening is higher for women who have been circumcised, and whether the provider will have a negative attitude toward a patient who has had the procedure [10–12]. Somali women often feel that their needs are not well met in the US health system [6, 13]. Somali men have some of the same barriers to screening and have comparably low screening rates for cancer [14]. Additionally, as men may also influence women in their decision-making processes, addressing the role of men in breast and cervical cancer uptake may also be important.

Despite the many complex barriers, Somali women have expressed interest in taking part in breast cancer screening [7], and the challenge remains to find ways to best support them in undergoing screening. The literature describes some of the barriers for breast and cervical cancer screening as originating from adherence to the Muslim faith [7], yet there is failure to address how these barriers might be related to interpretations of faith. When the understanding of faith is not taken into consideration, faith can be positioned as a barrier to overcome [15], rather than as a potential asset which could be used to encourage screening [16]. By integrating messages that are consistent with one’s faith alongside the many other factors that influence the uptake of breast and cervical cancer screening, a more comprehensive and culturally relevant approach can be used to address these disparities. One study focusing on Muslim women from Iran captures the idea that faith can even be a significant source of support in dealing with cancer [17]. Likewise, incorporating faith may be important in better addressing the concerns about screening held by Somali women and therefore in encouraging screening uptake [18]. In this sense, the integration of faith-based messages would support health screening behavior as one of multiple levels of intervention, consistent with the social ecological model [19, 20], which places attention on the need for multi-level interventions that draw on interpersonal, community, cultural, and structural barriers for engaging in screening [21]. This model has been identified as a useful framework to advance thinking beyond access to services and offers a relevant and important approach for comprehensive health change [22, 23].

One particular aspect of faith that serves as barrier to screening is a concern about modesty, because it is considered inappropriate to show private parts of the body to others, including medical providers and especially those of the opposite sex [7]. One way to address such concerns about modesty is to ensure that Muslim women have access to a female doctor [7], which can have a positive impact on cervical screening uptake for Somali women [4]. This indicates that where it is possible, the provision of same-gender providers can be important. In addition, some Muslims consider developing cancer to be a matter of fate or predestination, in that it is perceived to be due to the will of Allah [7, 15]. Therefore screening can be seen as an attempt to bypass the will of Allah. Additionally, men in the Somali community may feel that being adherent to the Muslim faith protects them from being susceptible to cancer [14]. Underestimation of cancer risk in immigrant populations [15, 24] may also negatively impact screening.

In this project, we partnered with an Imam in the community to develop faith-based messages focusing on ways in which Islam can be seen as encouraging cancer screening. The messages were developed collaboratively, based on existing data [18] and the Imam’s expertise. We then convened focus groups of Somali men and women to test the messages, delivered by the Imam via videos, as a potential asset in engaging women in screening. The messages drew on Islamic principles that are health promoting and encouraged a discussion of key areas. They offered faith-based perspectives on modesty, predestination and taking care of one’s self messages that are consistent with promoting screening and treatments for cancer. In the past some have viewed messages from their faith as barriers for screening uptake [7].

Exploring these research questions will help inform the potential use of faith-based messages as an asset to help address breast and cervical cancer screening disparities for Somali women. Such messages could potentially be used in a support of other approaches used to address the wider range of barriers facing women, such as in education and community outreach efforts.

## Methods

The authors worked collaboratively to develop the faith-based topics to be used to address modesty, predestination, and screening and treatment for breast and cervical cancer. The Imam (Imam Sharif Mohamed, research team member and co-author on this paper) developed the content of the messages based on his expertise as an Islamic scholar and faith leader, guided by previous work conducted by the research team. The messages were developed in a reflective process, during which the whole team met to draft and review the messages. The messages developed were based on Islamic principles and were presented both as a written document and as three professionally filmed video clips of the Imam (SM) discussing the messages.

### Procedures

We recruited 34 Somali women and 20 Somali men to a series of five focus groups, three with women only (two groups of eleven, and one of twelve) and two with men only (two groups of ten men each). We aimed to recruit between ten to twelve participants for each group. The focus groups were conducted in one urban mosque in Minneapolis, Minnesota. Participants were recruited through the mosque, and focus groups were conducted in Somali by a trained bilingual research assistant (Nimo Ahmed), as this was the preferred language of participants. Focus groups took place over approximately two hours, with breaks for prayers as necessary. The inclusion criteria were that participants identified as Somali and were willing to discuss the topics of breast and cervical cancer screening. The exclusion criteria included participants being unwilling to discuss, or distressed by discussing, breast or cervical cancer. This study was reviewed and approved by the University of Minnesota Institutional Review Board. Informed consent was obtained from individual participants through an IRB approval process of gaining verbal consent to participate.

Each focus group used a semi-structured guide to facilitate a discussion of participant views of breast and cervical cancer screening in general, then the group viewed each video clip and provided feedback on their views on the content on the clip. The clips’ core messages, which were delivered in Somali, are summarized in Table 1.

**Table 1:** Key aspects of faith-based messages targeting screening behavior

Faith-Based Topic	Key Messages
Predestination	• Screening does not bypass predestination • Seeking good health is consistent with faith • Treatment is also predestined and is there to be taken • Screening is an opportunity to be taken
The role of modesty	• Modesty of the heart is as important as of the body • It is preferred to see a doctor of the same gender • It is acceptable to see a doctor of the opposite gender • It is more important to seek a doctor who has knowledge and confidence in the health need at hand, than a doctor of the same gender. • In an emergency situation, it is important to first consider how to protect life, the concept of modesty remains but is secondary to protection of life.
Cancer screening and treatment	• Prevention of disease is a faithful and religious thing to do • Religion encourages care of the body along with the mind • Prevention is better than cure • Allah has entrusted you to care well for your life and body

### Development of messages

The development of the faith based messages was based on key findings from earlier work, where a number of barriers to breast and cervical cancer were identified, including concerns about modesty, predetermination and a lack of familiarity with breast or cervical cancer [18]. The messages that were particular to faith where identified and shared with a local Imam. The Imam drew on his expertise and texts (such as the Quran) to develop messages that addressed the faith based concerns, as they pertain to the idea of breast and cervical cancer screening. In that, these messages were not intended to be comprehensive messages on matters of faith, but targeted brief messages that explored how faith related to these specific concerns as they relate to breast and cervical cancer screening behaviors. These messages were drafted, and reviewed by the study team. Each team member engaged in discussion about the messages, and the faith based concerns they were intended to address, until a consensus was reached about the message content. This was done initially as a written document, which was subsequently used to as the basis for series of short video messages, delivered in Somali by Imam Sharif Mohamed. Imam Sharif Mohamed is the founder of the first mosque led by Somali community in MN. It is one the larger mosques in MN and is one of the most populated due to its age and centrality to a large Muslim population in the neighborhood. It is based in the heart of the community and is well known to most East African Muslim immigrants who live in the area. He also Chair to the Islamic League of Somali Scholars which is a national, umbrella organization. His standing as a scholar is generally unquestioned among Muslims in Minnesota.

After each video clip, participants were asked for their views on the contents of the clips and how they may or may not influence their own views on breast and cervical cancer screening.

#### Qualitative data analysis

All focus groups were conducted in Somali, recorded using digital voice recorders and were simultaneously translated and transcribed verbatim by professional bilingual transcriptionists. Transcripts were analyzed using NVivo10 [25] to facilitate the analysis of qualitative data. Data were analyzed using the social constructivist version of grounded theory, through which themes and subthemes were identified in the data [26, 27]. The social constructivist approach does not aim to develop novel theories from data alone, rather it is an iterative process of review that allows consideration of existing knowledge and literature that can be drawn upon in the analysis process [26, 27]. The research team met throughout the analysis process to review the emerging themes and discuss areas of agreement or divergence until consensus was reached.

#### Participant demographics

The 34 women and 20 men who participated in the five focus groups represented a range of demographics. However, the male participants were more likely to be in full-time employment (60%), to have college-level education (35%), and to have lived in the US for longer than 10 years (70%) than the female participants (12%, 18%, and 44%, respectively) (Table 2).

**Table 2:** Participant Demographics

	Female Focus groups (*N* = 34)	Male Focus groups (*N* = 20)
Work status		
Full time	12% (4)	60% (12)
Part time	20% (7)	10% (2)
Did not work outside the home	53% (18)	25% (5)
Other	15% (5)	5% (1)
Schooling		
None	32% (11)	15% (3)
Middle or high school	50% (17)	50% (10)
College	18% (6)	35% (7)
Duration of residence in US (y)		
≤ 5	12% (4)	25% (5)
6–10	38% (13)	5% (1)
> 10	44% (15)	70% (14)
Not answered	6% (2)	
Age (y)		
18–29	11.7% (4)	10% (2)
30–39	29.4% (10)	55% (11)
40–49	11.7% (4)	10% (2)
50–59	17.6% (6)	0% (0)
60 or older	29.4% (10)	25% (5)

Participants had varying histories of cancer screening. Of the male participants, 90% reported having no experience with cancer screening of any sort (Table 3), although they were also a notably younger sample than the female participants (Table 2). Of the female participants, 41% had undergone a mammogram and 59% had experienced a pap test, and 68% were planning to have screening in the future (Table 4). Only 20% of male participants were intending to engage in future screening.

**Table 3:** Male participant’s previous experience with cancer screening

Male Focus groups (*N* = 20)	
Have you ever had any type of cancer screening?	
Yes	0% (0)
No	90% (18)
Unsure	10% (2)
Have the women in your family ever had cancer screening?	
Yes	0% (0)
No	90% (18)
Unsure	10% (2)
Are you planning to have any type of cancer screening in the future?	
Yes	20% (4)
No	25% (5)
Unsure	55% (11)

**Table 4:** Female participants’ previous experience with cancer screening

Female Focus groups (*N* = 34)	
Have you ever had a mammogram?	
Yes	41% (14)
No	56% (19)
Unsure	3% (1)
Have you ever had a pap test?	
Yes	59% (20)
No	38% (13)
Unsure	3% (1)
Are you planning to have either a mammogram or a pap smear in the future?	
Yes	68% (23)
No	20% (7)
Unsure	12% (4)

## Results

Three main themes emerged in the analysis: 1) the experience of breast and cervical cancer screening; 2) the relationship between faith and screening; and 3) views on the faith-based messages on the topics of predestination, modesty, and screening and treatment.

**Table:** 

Main Theme	Sub-theme
The experience of breast and cervical cancer screening	Varying understanding of cancer
	Negative experiences of screening
	Cancer viewed as deadly
The relationship between faith and screening	Attempt to predict onset of disease
	Seeking knowledge about health
	Modesty
	Death is inevitable
	Health and faith can coexist
Views on faith based messages	Views on predestination
	Views on modesty
	Views on treatment

### Experience of breast and cervical cancer screening

Participants had **varying understandings** about breast and cervical cancer. Some described knowing family members with cancer, and one female participant described having had cancer herself (uterine cancer). Many participants shared that they knew what cancer was, but in their view it was something that was almost always described as serious or deadly. In the focus groups with women, participants were asked to share their own experiences with breast and cervical cancer screening and many had **negative experiences of screening**. Many had been asked to undergo screening tests, and some described they had done the screening, but some women described actively avoiding screening tests. One woman avoided the tests by avoiding the doctor entirely.

The doctor always called me, but I ignored his calls and each time cheated them by telling them I am busy, always, will call back, etc. And as a result of that, I have never had any examinations whether breast or pap smear. (Women’s Focus Group 1)

The reasons for avoiding the tests were fear of the mammography machine, negative experiences with testing in the past, and experiences of pain in undergoing screening. Some were concerned that the mammography machine would cause damage to the breast and were not satisfied with provider reassurances. Some women described that they had no symptoms of disease or illness, and therefore they did not need to undergo testing for either breast or cervical cancer.

I don’t know why I need it if I have no sickness. People know when they are sick and when they need help. So my feeling is my breast and cervix are healthy, thank God. All I have is a bit of hypertension and arthritis. Therefore, I told the doctor I don’t need any of the tests. (Women’s Focus Group 3)

The men in the study also shared their concerns that **cancer was a serious and deadly disease**, but most did not know if the women in their families had undergone screening for cancer. Despite this, some men talked about mothers and wives having had testing and described being supportive of that. Male participants also described that the symptoms of illness would need to be present in order to feel they would need tests that would screen for cancer.

### Relationship between faith and screening

Participants were asked to share how faith might impact their views on screening for breast and cervical cancer. For some, there were concerns that screening was **an attempt to predict the onset of disease**. To attempt to make predictions about what might happen to an individual was seen as trying to predict Allah’s will, which can only be known to Allah. In addition, some described their view that if Allah was the only one that would know if someone would develop cancer, that would make preventive screening irrelevant.

He repeatedly asked me to have the test, but I kept telling him I don’t need it. He told me are you sure? I said yes. He then asked me, what if we do it for prevention? I said Allah will protect me. For what is coming, no one knows but Allah. For now, I know I don’t need it. (Women’s Focus Group 3)

Yet for other participants, faith was seen to encourage **seeking knowledge about health**, and as being supportive of undergoing cancer screening.

As Muslims, we are asked not to show suspicion about what is good about your health or for your health. The religion says **“**shaki waha kafican yagin” — knowing is better than suspicion. (Women’s Focus Group 2)

Concerns about **modesty** were also mentioned by participants, who shared that modesty must be observed in relation to receiving health care. This was particularly important in relation to the gender and, for some, religion of the provider. Many women expressed a preference for female providers and Muslim providers, although it was considered acceptable to see non-Muslims or men under particular conditions, such as urgent health problems. However others felt that faith and medicine were compatible aspects of health that one could engage simultaneously.

No religion prohibits seeing a doctor. For a disease, cancer in particular, we need a doctor’s help and treatment. Of course we also pray to God. So I see we can do both at the same time. (Men’s Focus Group 1)

Should cancer be identified through screening, there were a range of views about how faith would influence decisions about care and treatment. Some felt that **death would be inevitable**, and treatment wouldn’t be able to stop that. Some felt that becoming ill and dying is the will of Allah, and it would be right to let the illness run the course intended by Allah.

Others felt that **health care and faith could exist together**, and it was part of faith to seek the best care and treatment available. For some this was expressed by seeing Allah acting through the doctors and through the treatments, in a fully integrated experience of faith and medicine working together. However treatment was seen by some as existing in the context of having symptoms, such as pain. Having treatment for those symptoms was seen as acceptable. Some also said that their faith influenced them to have a view that all life is precious, and all must be done to preserve life, including seeing doctors. Ultimately the success of treatment was seen to be in the hands of Allah, but seeking treatment was seen as consistent with faith.

Everything that happens to us is within Allah’s command. I believe the life of a human being is expensive and precious. We have to consider everything within our power. We seek treatment from Quran recitation. We seek treatment from doctors. We have to try all we can to protect a human’s life. We also have to acknowledge that everything happens because of Allah’s will. So we have to seek treatment knowing that in the end, it is God’s decision to make. (Women’s Focus Group 3)

### Views on faith-based messages

Three video clips drawing on faith-based messages that promoted screening were presented half-way through the focus groups, eliciting discussion on how the content of the videos influenced the views of participants on breast and cervical cancer screening.

#### Predestination

After viewing the video, all of the focus groups showed high levels of agreement with the messages that screening is not in conflict with predestination. For some participants, the messages reinforced what they already believed. Additionally, some described how Allah can also work to treat people through medicine or medical providers. This was taken to mean that screening, treatment, and care also come from Allah and are therefore consistent with faith. Some spoke of the medical care available to them in the US as an opportunity to access tests, facilities, and treatments that wouldn’t have been available in Somalia. The opportunity was described as a positive thing not to be wasted, but to be taken up through participation in screening.

I agree with the video message by the sheikh. Indeed, we all need to check ourselves and take advantage of the treatment and health facilities we have. There are others who are not privileged as we are and have no health care. We need to take advantage of this opportunity. (Women’s Focus Group 1)

For some, they felt they learned new information from the video clips. This may be particularly the case for participants who held views that screening was in conflict with predestination. As a regarded community leader, the Imam’s views were described as credible and respected. New information presented was therefore seen as relevant and accurate. Some described changing their views and their intended future screening behaviors on the basis of viewing the messages.

We were talking about a disease. But the sheikh has reconciled the religious provisions and told us that religion permits us to do tests. So what has changed is that I now know religion allows us to have tests. (Men’s Focus Group 2)

Yes, the video message is very useful to me and I have now changed my opinion. From now on, I will seek more treatment and comply with prevention. (Women’s Focus Group 1)*.*

#### The role of modesty

There were high levels of agreement that modesty was important and could be maintained while having screening and treatment for breast and cervical cancer. Generally, participants agreed that it was preferable to see a provider of the same gender, with some also preferring the provider to be Muslim. However, there was wide agreement that under the right circumstances there were no constraints on the gender of the provider. Those circumstances were described as being when there wasn’t another choice available, or it was an emergency.

I am commending the sheikh’s message for this positive message. Actually, all doctors have good skills and education to attend to everyone from all genders. We will comply but also say females should express a preference for female doctors. In the absence of female doctor, then the male doctor can attend to them. (Women’s Focus Group 1)

Both male and female participants described a shift in views to be more prepared to accept a doctor of a different gender if needed in order to have care. Some participants reported finding the messages to be reassuring and encouraging, rather than new knowledge. Such encouragement inspired increased confidence in addressing these issues when seeing doctors for screening.

I have always known our religion is nurturing, and I agree with the decisions. The video message has been very encouraging and reassuring. (Women’s Focus Group 1)

Participants described modesty as being essential to faith, but described how there is modesty of the body, and modesty of the heart. In relation to screening, having modesty of the heart was important and meant that the need to be modest could still be preserved, even in the context of provider interactions where private parts of the body might be seen by the provider.

I believe the question is about modesty. The sheikh told us shyness and good character is from your heart. If you have a good heart and you have no sexual intention, then the doctor’s examination of your private parts should not be a problem. (Men’s Focus Group 1)

#### Cancer screening and treatment

Some participants described that the idea of cancer screening was new to them, and not something they felt they had necessarily had understood well. Some felt that cancer was a US disease, and not one they had been aware of in Africa. As a result, having screening felt very uncomfortable, particularly when the purpose of the tests wasn’t clear. Participants in both groups described the faith-based messages as helping to clarify the value of screening, and therefore increasing the sense of the importance of screening.

We Somalis do not practice prevention, but now we know this is important. (Women’s Focus Group 2)

Not only was cancer a new idea, but one male participant described how the messages had challenged his fears that screening could cause cancer, or plant the disease within him. He described his view as one he felt was commonly held within the community, and that the faith-based messages had encouraged him to reexamine this. He described the faith-based messages changing his knowledge, and felt that more sharing of these messages was needed in the community.

The last-played clip and the program changed me a lot. Before this, I had a bad idea about checkups or going to the doctor without actually being sick. I used to fear that if I went there when I was not sick, the disease would be planted in me. I am not joking or lying.… A lot of people I know have the same phobia about seeing a doctor for prevention’s sake. Now, this program changed my views. From now on I won’t be afraid of using prevention by seeing a doctor or by examining myself. (Men’s Focus Group 2)

There was also a change in how the role of symptoms was viewed by many participants. Both men and women talked about only using clinic services when they were unwell or experiencing pain. Some expressed having changed their views after viewing the faith-based messages and would consider undergoing tests even when they did not feel unwell. Men and women described a desire to share the messages with others in their families and community. Many described feeling that the faith-based messages had influenced how they viewed screening, and that they were now feeling more open to taking up opportunities to be screened for cancer.

Whatever my initial thinking in the past, my opinion has changed now and I am ready to change my thinking. I am ready to accept examinations. I have never wanted to be part of prevention checkups but now my thoughts are changed and I am ready for it now. (Women’s Focus Group 1)

Some men talked about how they wished to support the women in their families in seeking breast and cervical cancer screening. Some reflected they felt inspired by the messages and wished for a broader discussion in the community about screening; about prevention in general for all, including a consideration of cancers that impact men; and about the support of those who may develop cancer.

Truth is, I am changed. We were watching a program about our lives and health. And this disease, as told, happens more to women. It also happens to men. What I learned is the importance of prevention and checkups. Also, not just about ourselves, we need to help each other and support those who have the disease. And yes, we will share these messages with our family, relatives, friends, coworkers, and the community. (Men’s Focus Group 2)

## Discussion

In this work we set out to test the acceptability of faith-based messages aimed at ameliorating concerns among Somali women and men that cancer screening conflicts with the religious value of maintaining modesty and the concept of predestination, as well as promoting screening and treatment for cancer. Both Somali women and men had an overwhelmingly positive response to the faith-based messages promoting breast and cervical cancer screening. The faith-based messages appeared to reinforce the views of those who were already positively inclined toward screening, with participants describing increased confidence to engage in screening. For those who had reservations about screening, there was a great deal of feedback that the faith-based messages had influenced their views considerably. This indicates there may be substantial benefits to including faith-based messaging as a way to enhance interventions that focus on individual behavior change. Future research that assesses uptake of screening by participants will be needed to fully test the impact on screening behavior.

Some Muslims may see Allah’s (God’s) will as being expressed through human agents, such as Imams or health care providers, or through other interpersonal relationships [28]. In this sense, seeing your provider for cancer screening, or having treatment, may also be considered to be following God’s will. Participants in this study described this by explaining that God may have given them the opportunity for screening or treatment, but the outcome would still be predetermined by Allah. This means that engaging in these interventions does not mean a Somali patient is trying to avoid their destiny, but the screening and treatment can be reconciled with faith and be compatible with predestination. This suggests that faith-based interventions might be uniquely placed to address barriers to screening across the various layers of the social ecological model [19, 20], because considering the role of faith in a wide range of cancer prevention efforts could help draw on faith as an asset on many different levels, including efforts to encourage individual behavior change as well as changing community wide attitudes, or clinic awareness.

Imams may play an important role in providing health promotion messages to their community, including the value of undertaking preventive screenings and treatment [29, 30]. In this research, messages shared by an Imam were seen to be acceptable and influential because they were being delivered by a faith leader. The participants in the research indicated that messages seemed to have encouraged them to view screening and treatment for cancer as something that is consistent with their faith. It may be useful to incorporate faith based messages into interventions that aim to address the broader range of barriers facing Somali women, such as health literacy [6], knowledge [7, 8] and concerns about female circumcision [8, 9].

This work is novel in that both men and women were very engaged, although in separate settings, in talking about breast and cervical cancer screening in the Somali immigrant community. Men talked about the value of learning about breast and cervical cancer, and expressed enthusiasm for being supportive of women in their families undergoing screening. Men also talked about the value of thinking about cancer in their own lives and applied learning about cancer to their own experience. This indicates there may also be great potential to tailor faith-based messages for men, or to create general messages about cancer for both genders. Women in the focus groups also voiced their desire for men to be engaged in discussions about cancer in general, and breast and cervical cancer in particular. Participants expressed a strong desire to share the messages with their own families, workplaces, and communities. This indicates that work to improve screening that engages families as well as individuals in conversations about cancer might be a useful approach in the Somali immigrant community.

There are a number of limitations in this work, one of which is that we did not test the impact on actual screening behavior. Future work that collects the incidence of screening events will be important. The sample may also have been subject to bias through self-selection, in that participants who were prepared to participate in the project may have been more willing to examine their views than those who chose not to participate. Additionally, the Somali immigrant community is diverse, and a larger sample might represent greater diversity of community members. In particular, there may be differences in response to this work based on age, or level of religiosity, and that was not explored in this research.

## Conclusion

This study has shown that the Somali immigrant women and men in this study were responsive to faith-based messages that addressed topics of predestination, modesty, and the use of screening and treatment. These messages could therefore be used as an asset to promote breast and cervical cancer screening. Future efforts to address cancer disparities for Somali immigrants should avoid seeing faith as a barrier, but rather look to integrate faith as an asset in promoting cancer screening.

## Abbreviations

IRB: Institutional Review Board
MN: Minnesota
